# Model-based in silico analysis of the PI3K/Akt pathway: the elucidation of cross-talk between diabetes and breast cancer

**DOI:** 10.7717/peerj.5917

**Published:** 2018-11-09

**Authors:** Sammia Rehman, Ayesha Obaid, Anam Naz, Amjad Ali, Shahzina Kanwal, Jamil Ahmad

**Affiliations:** 1 Atta-ur-Rahman School of Applied Biosciences (ASAB), National University of Science and Technology, Islamabad, Pakistan; 2 Guangzhou Institutes of Biomedicine and Health, Guangzhou, China; 3 Research Center for Modeling & Simulation (RCMS), National University of Sciences and Technology, Islamabad, Pakistan

**Keywords:** Petri net, Breast cancer, OGT, PI3K/Akt pathway, Insulin resistance

## Abstract

**Background:**

A positive association between diabetes and breast cancer has been identified by various epidemiological and clinical studies. However, the possible molecular interactions between the two heterogeneous diseases have not been fully determined yet. There are several underlying mechanisms which may increase the risk of breast cancer in diabetic patients.

**Introduction:**

In this study, we focused on the role of O-GlcNAc transferase (OGT) enzyme in the regulation of phosphatidylinositol-3 kinase (PI3K) pathway through activation/deactivation of Akt protein. The efficiency of insulin signaling in adipocytes is reduced as a result of OGT overexpression which further attenuates Akt signaling; as a result, the efficiency of insulin signaling is reduced by downregulation of insulin-responsive genes. On the other hand, increased expression of OGT results in Akt activation in breast cancer cells, leading to enhanced cell proliferation and inhibition of the apoptosis. However, the interplay amongst these signaling pathways is still under investigation.

**Methods:**

In this study, we used Petri nets (PNs) to model and investigate the role of PI3K and OGT pathways, acting as key players in crosstalk between diabetes and breast cancer, resulting in progression of these chronic diseases. Moreover, in silico perturbation experiments were applied on the model to analyze the effects of anti-cancer agents (shRNA and BZX) and anti-diabetic drug (Metformin) on the system.

**Results:**

Our PN model reflects the alterations in protein expression and behavior and the correlation between breast cancer and diabetes. The analysis proposed two combination therapies to combat breast cancer progression in diabetic patients including combination of OGTmRNA silencing and OGT inhibitor (BZX) as first combination and BZX and Metformin as the second.

**Conclusion:**

The PN model verified that alterations in O-GlcNAc signaling affect both insulin resistance and breast cancer. Moreover, the combination therapy for breast cancer patients consisting of anti-diabetic drugs such as Metformin along with OGT inhibitors, for example BZX, can produce better treatment regimens.

## Introduction

Recent epidemiological studies suggest that approximately 400 million people have Type II diabetes worldwide (Guariguata et al., 2014). Several causes can increase the risk of Type II diabetes such as genetic predisposition, behavioral and environmental risk factors. Recently, diabetes has been related to increased risk of cancer, among them, breast cancer is the most common (Larsson, Mantzoros & Wolk, 2007). According to Wolf et al. (2005), diabetic patients have 25% increased the risk of breast cancer in cohort studies (Wolf et al., 2005). Along with other risk factors such as obesity and hyperglycemia, insulin resistance in Type II diabetic patients enhances the possibility of cancer and cancer-related mortalities (Gapstur et al., 2001). In addition to this, breast cancer is known to be the most common malignant neoplasm among women of developed countries (Torre et al., 2015). This risk is increasing day by day within developing countries including Pakistan at an alarming rate, making it second to Non-Arab Israeli women. One in nine women develop breast cancer at some stage of their life in Pakistan (Sohail & Alam, 2007). Risk factors for breast cancer include age, hormonal factors, obesity, benign breast disease, family history and genetics (Key, Verkasalo & Banks, 2001). Diabetes has now also been linked as a major risk factor. Both of these chronic diseases (diabetes and breast cancer) are positively associated, heterogeneous in nature and multi factorial in origin (Joshi, Liu & Turner, 2015).

Cancer cells exhibit an alteration in cellular energetics processes and are well known for their addiction to glucose. Hyperglycemia is a strong inducer of proliferation as well as of enhanced progression of cancer cells. It is usually caused by hyperinsulinemia, a condition in which insulin rises above normal levels (10–20 mcU/ml) due to insulin resistance (Modan et al., 1985). Thus, the abnormality in insulin signaling is one of the common associations between Type II diabetes and breast cancer (Ferroni et al., 2015). Increased glucose level and deregulated metabolism showed a well-established association with insulin resistance (Jellinger, 2007). Therefore, it is important to decipher the role of different metabolic pathways in this context, particularly glucose metabolic pathways including Hexosamine Biosynthetic Pathway (HBP). 3–5% of glucose entering into cells led to HBP, producing UDP-GlcNAc as an end product, a substrate used by O-linked N-acetyl glucosamine transferase OGT. OGT is the enzyme responsible for adding O-GlcNAc moiety on serine and threonine residues of nuclear and cytoplasmic proteins in a reversible post-translation modification called O-GlcNAcylation and is antagonized by another enzyme called O-GlcNAcase (OGA). Several studies reported the role of HBP in alteration of insulin signaling pathway but there exist certain controversies amongst the studies regarding the exact mechanism (Vosseller et al., 2002). It is known that the increased level of O-GlcNAc on various proteins involved in the phosphatidylinositol-3 kinase (PI3K) pathway results in worsening diabetic complications (Akimoto et al., 2015). In 2008, Yang et al. (2008) found an association between OGT and PI3 Kinase, which can cause alternation in insulin-responsive signaling, leading to attenuation of insulin pathways. Similarly, OGT has been shown to play an important role in tumor progression and metastasis. In 2010, Caldwell et al. (2010) reported increased OGT gene expression and enhanced level of O-GlycNAcylation in cancer cell lines compared to normal cell lines, which are associated with invasive properties of breast cancer cells in vivo. Additionally, enhanced OGT gene expression has been observed with the increase in tumor grade, implicating significance of OGT in cancer progression (Krześlak et al., 2012). Emerging significance of OGT in both of these chronic diseases, that is, diabetes and cancer necessitates modeling the dynamics of the PI3K/Akt pathway in a holistic manner for a better understanding of OGT function in an insulin resistant as well as in hyperglycemic cellular state during diabetes and breast cancer. As cellular signaling networks are highly complex in nature, the analysis of each and every molecule or protein becomes difficult, however, systems biology approaches provide a platform to analyze such complex systems holistically. It enables us to understand how bio molecular networks function as a dynamic system.

Over the past decade, computational tools have been vigorously used to analyze biological systems in real time. The core of systems biology consists of methods to build an integrative and coherent picture. Computational modeling of systems not only helps in investigating the relationships and behavior of elements involved in a biological system but also explains how the system functions as a whole. Moreover, diagrammatic models summarizing biological systems improve mechanistic understanding of the observations. Computational tools such as Ordinary Diﬀerential Equations based models have been applied to study various biological systems. Recently, the role of OGT enzyme in cancer progression has been studied by Saeed et al. (2016) through computational modeling in which formal modeling has been applied. An established technique for modeling of the biological systems is the Petri nets (PNs). PNs consider concurrency of a system that is vital to model biological systems. Recently, PNs have widely been used to study and model metabolic pathways (Reddy, Mavrovouniotis & Liebman, 1993). PNs can analyze large-scale networks in order to predict cellular behavior based on cell signaling without requiring an in-depth knowledge of the parameters of signaling networks (Ruths et al., 2008). It is based solely on network’s connectivity and aids in studying trends and activity levels of molecules present within a cell signaling cascade in response to a stimulus.

### Our contribution

For the sake of simplification, the PI3K pathway has been divided into three states; normal intact pathway; altered pathway in adipocytes and hyperactivated pathway in breast cancer. In breast cancer, the PI3K pathway has been studied under the hyperglycemic condition to understand the consequences of increased O-GlcNAcylation on proteins involved in the PI3K pathway. Moreover, the effect of OGT mRNA inhibition, OGT protein inhibition, and Metformin was studied in silico on various cell processes in order to gain clarity on the significance of OGT function in cellular processes. When subjected to specific perturbations, the results reflect the alterations in protein expression and behavior. We analyzed how cellular function is altered as a cell is under a hyperglycemic condition and upon OGT inhibition through various interventions, these functions, for example, cancer cell proliferation and survival is reduced. The predictions made through this study can further be validated in the wet lab. This study is not only exhibiting significance of OGT regulation in these diseases but it also helped us to predict that OGT inhibition might reduce cancer cell proliferation and its inhibition in combination to Metformin can reverse breast cancer progression to a significant extent.

## Materials and Methods

### Petri net modeling

Petri nets having a simple and flexible framework are based on a graphical and mathematical formalism that is highly applicable to model and analyze asynchronous, concurrent and distributed systems (David & Alla, 1992; Peterson, 1981; Reisig, 1985). Thus, it has been successfully applied in various domains and studies, such as biochemical processes, biological pathways/networks, industrial mechanisms, software analysis etc. (David & Alla, 2010). A PN contains two sets of vertices called places and transitions. Resources of the system are depicted by places while the events that change the resource state are represented by transitions. These places and transitions are connected through edges. A place holds tokens that might define for example the number of molecules involved in the system. Edges move the tokens causing a change in the system through a transition.

#### Standard petri nets

**Definition:**

A PN is a bipartite graph consisting of two sets of vertices, places and transitions.

Formally A standard Petri net is a quadruple N = (P, T, f, m0),

where:

P, T are finite, non-empty, disjoint sets. P is the set of places. T is the set of transitions.

*f* : ((*P* × *T*)) ∪ ((*T* × *P*)) → ℕ *defines the set of directed arcs, weighted by non-negative integer values.*

*m*_0_ : *P* → ℕ_0_ *gives the initial marking* (*Heiner et al., 2012*).

A place describes a resource or an entity (for example, proteins, DNA, RNA etc.) and its state (number of entity present, relative level, cellular concentration etc.). In comparison, a transition describes any process occurring in the system. In a PN, the edges or arcs always connect vertices from two distinct sets only, that is, places connect to transitions and vice versa. The weight of an arc is equal to 1 by default and it represents the multiplicity. Places present before transitions are called―input places (source) whereas places after transitions are―output places (sink) for that specific event. An arc with a hollow dot at its head represents an inhibitory arc. The function of an inhibitory arc is suppression of token flow as it stops the firing of a transition. Tokens are denoted as numbers or dots within a place in a PN. They are variable and represent states of entities (Heiner et al., 2012). Tokens, in particular, signify relative concentration levels of entities like RNA, proteins, ions, organic and inorganic molecules in a biological system (Li et al., 2006). Marking represents the state of the system based on the presence of tokens in a particular state at that instance. In a dynamic system, marking evolves with time as the tokens flow in the model. All the input places must have tokens to fire a transition. In accordance with respective arc multiplicities, the number of tokens withdrawn from input places and deposited to output place after a transition has been fired (Heiner et al., 2012).

### Non-parametric strategy for petri net modeling

A number of studies have employed PN approaches to model gene regulatory networks and cell signaling pathways (Awan et al., 2017; Li et al., 2006; Naz et al., 2017; Sackmann, Heiner & Koch, 2006). Due to limited analytical resources in conventional experimental approaches, it is difficult to analyze kinetic parameters of each and every biological reaction in a system. Therefore, a non-parametric strategy, as formulated by Ruths et al. (2008), was implemented in our study to explore the dynamics of cell-specific signaling pathways employing PN approaches. The PN model is based on the assumption that the signaling network connectivity is the most significant determinant of signal propagation (Li et al., 2006). Therefore, changes in the activity levels of the proteins within a particular signaling pathway correlated with their abstract quantities, which depicts the relative change only and are represented in the PN model by token number (Ruths et al., 2008).

### Pathway abstraction

**Figure 1:**
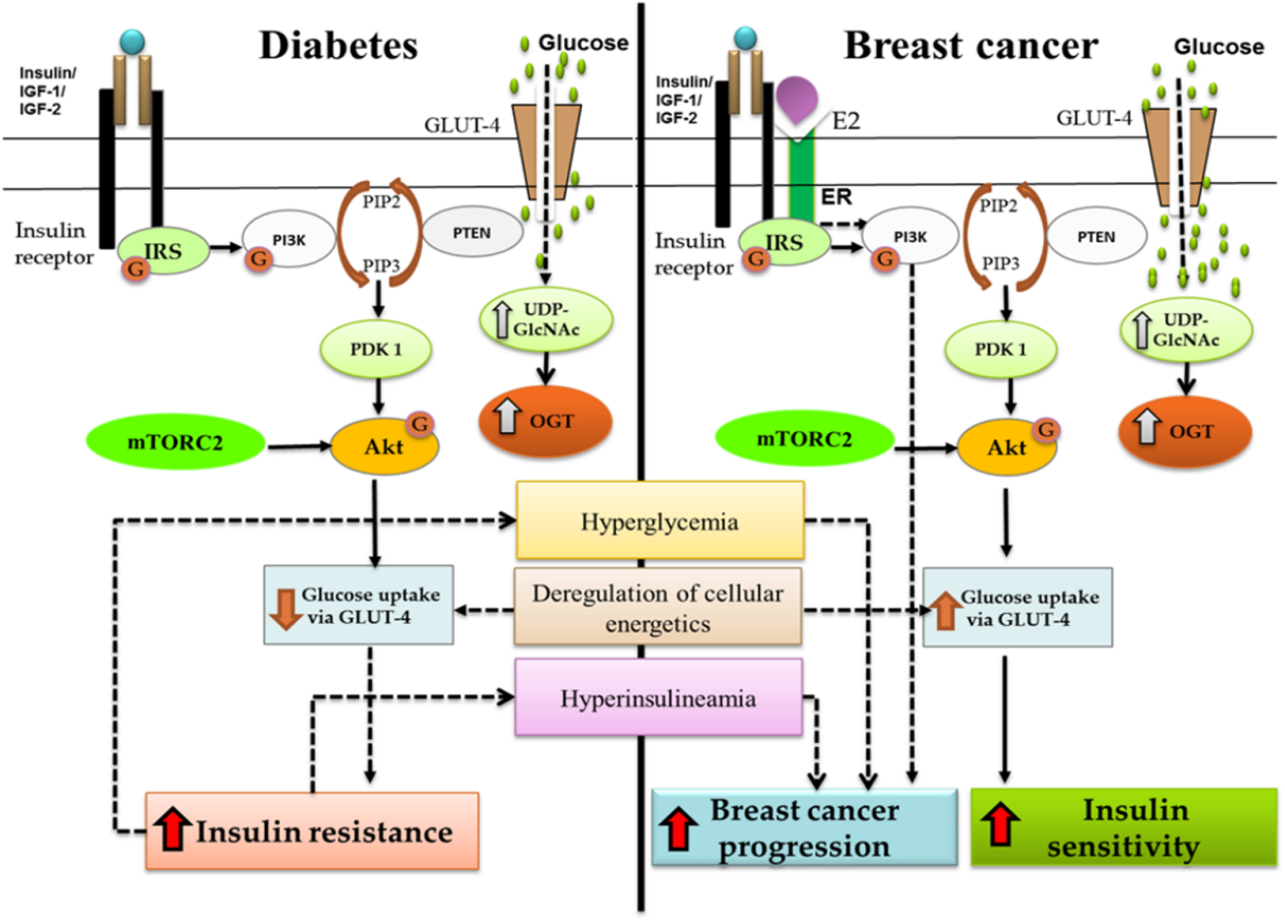
Crosstalk between diabetes and breast cancer. Binding of insulin molecule to insulin receptor leads to auto-phosphorylation of tyrosine residues present on insulin receptor. A phosphatidylinositol-3 kinase (PI3K) is translocated to the cell membrane and activated. As a result, phosphatidylinositol-3,4,5-triphosphate (PIP3) is produced, which recruits Akt. The interaction of PIP3 with the PH domain of Akt induces conformational changes in Akt, thereby exposing the two main phosphorylation sites at T308 and S473. Phosphorylation at T308 and S473 by protein serine/threonine kinase 3′-phosphoinositide-dependent kinase 1 (PDK1) and mTOR Complex 2 (mTORC2) respectively, is required for maximal Akt activation. Activated Akt can have a number of downstream effects such as glucose uptake via GLUT-4, regulation of apoptosis, promotion of cell survival and protein synthesis. PI3K/Akt pathway forms the junction for cross-talk between diabetes and breast cancer. As shown in the figure, increased insulin resistance leads to hyperglycemia and hyperinsulinemia which further leads to breast cancer progression. OGT is overexpressed in both the systems and deregulation of cellular energetics effects GLUT-4 expression causing either insulin resistance (diabetes) or increased insulin sensitivity (breast cancer).

The complex pathway taken from KEGG database (pathway ID hsa04910) Kanehisa et al. (2017) was analyzed and key proteins (IRS-1, Akt and GLUT-4) that play a central part in both diabetes and breast cancer were isolated (Fig. 1). Since the pathway under study, involved a number of proteins regulating the system, we restricted the pathway to key proteins only. After restricting the key proteins, we applied the strategy to carry out abstraction of PI3K/Akt pathway as explained by Paracha et al. (2014). Figure 1 depicts the selected key proteins considered in this study. The extracted pathway was then subjected to qualitative PN modeling.

### Construction of the petri net

**Figure 2:**
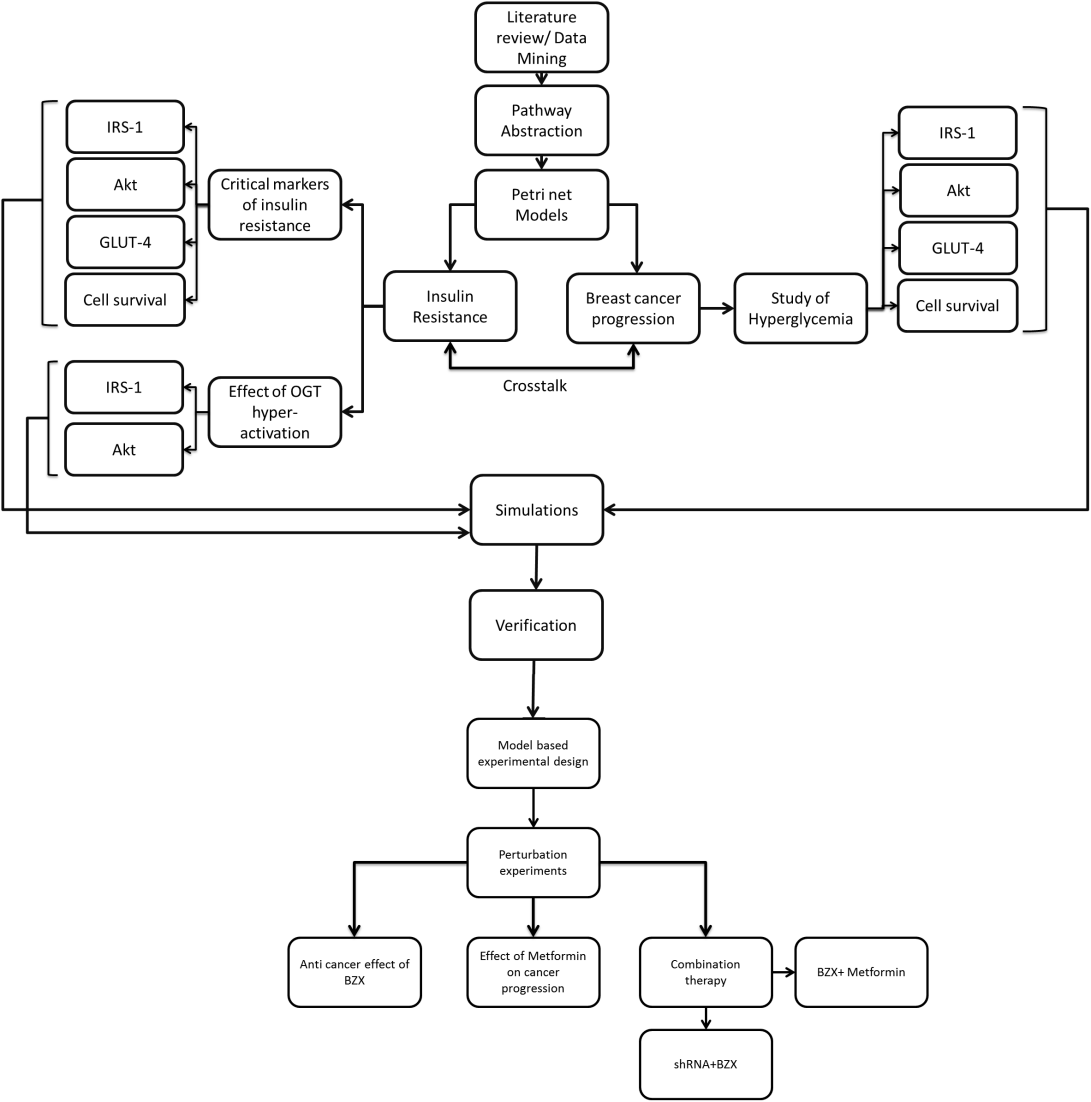
Flowchart explaining model-based study design. After collecting data from the literature, PI3K/Akt pathway was reduced such that key proteins represent the ultimate effector functions and regulations. It was next subjected to PN modeling. Markers critical for insulin resistance, the effect of OGT hyper-activation and breast cancer progression under hyperglycemia were studied in diabetic and breast cancer system. Simulations were run and results are verified through literature studies to ensure correctness of our model. To study the efficacy of OGT inhibition in breast cancer in silico perturbation experiments were carried out using shRNA- microRNA inhibitor (OGTi), BZX- A covalent/suicide OGT protein inhibitor, Metformin-A renowned anti-diabetic drug. We also checked the combination effect of shRNA + BZX and BZX + Metformin in our model.

The steps involved in the PN model generation include literature survey to extract the critical factors involved in insulin resistance and breast cancer with and without hyperglycemia; pathway abstraction; generation of PN model and its analysis. The study design has been described in Fig. 2. A cell signaling pathway is activated when a ligand interacts with corresponding receptors present on the cell surface. In turn, it activates downstream proteins through modifications like phosphorylation, de-phosphorylation or interaction with proteins. To model such a complex and dynamic pathway a modeling strategy was formulated that well-suited the network topology of the disease model (diabetes and breast cancer). In the designed PN model places in a circle represent the proteins and genes (e.g., insulin receptor, ligands, enzymes, transport proteins, genes etc.) involved in the PI3K/Akt pathway while the transitions in square represent the processes like interactions or reactions occurring among the places (e.g., formation of complex, chemical reactions, post-translational modification, transport processes etc.). Certain entities have been represented with colors for clarity, for example, blue places show entities IRS1, Akt, GLUT4 and cell survival. Other entities include OGA (Orange), OGT (Red), increased glucose (Pink), shRNA (Light blue), BZX (Green) and Metformin (Purple). The markings of continuous places are real numbers and the firing of transitions is a continuous process. All the arcs have a weight equal to 1 except for those mentioned otherwise. Moreover, inhibitory arcs are used to show inhibitory effects of the anti-diabetic drug (Metformin) on cellular processes. Our model depicts source transitions as the availability or synthesis of proteins/ drugs in the pathway while sink transitions represent the decay or dissociation of entities exiting the system.

The concentration level of an entity (protein, DNA, RNA etc.) is represented by tokens. In order to perform simulations, it is important to indicate the availability of entities in a biological system. Therefore, arbitrary initial values were assigned as tokens in the PI3K/Akt pathway that corresponded to an initial state of entities in the cell. In our study, PN was designed to understand relative activity change (up-regulation/down-regulation) and not the exact measurement of the protein concentration/parameters within the PI3K/Akt signaling pathway. In the present study, PN model generation and simulations were run using Snoopy version 2.0 (Heiner et al., 2012).

### Perturbation experiments

Perturbation biology is a useful tool to study the effects of therapeutic drugs or protein inhibitors for further clinical research. If successful, the proposed drugs or inhibitors can be implemented in clinical settings followed by clinical trials. PNs allow the use of such experiments to predict the specific behaviors on exposure to such external stimuli.

Based on data found in the literature, we constructed a cell-type specific signaling model that linked drug-induced perturbations and cellular mechanisms. In silico perturbation experiments, as shown in Fig. 2, were designed to study RNA interference by shRNA and protein inhibition by OGT inhibitor (BZX) of OGT. The individual effect of anti-diabetic drug Metformin was studied on breast cancer cell proliferation. Moreover, the effect of combinatorial therapeutics for ameliorating breasts cancer, that is, shRNA (against OGTmRNA) + BZX and BZX + Metformin was also investigated. The PN models successfully captured the signaling events and determined responses to combinatorial interventions that were previously untested.

### Model verification through simulations

To verify the designed pathway, our methodology utilized the simulative property of PN. A PN carries out simulations of place/ transition network with the token flow. It can help us to predict the dynamics of the model with time. Analysis of a state space subset or all the possible state spaces can be done through simulation runs. To enhance the model accuracy and narrow down the parameter search space, prior pathway information was extracted from signaling databases. All the properties that were studied in our PN model were compared with wet lab experiments carried out previously. Our PN model was verified when the selected properties such as overexpression of receptors and proteins matched the experimental studies.

### Understanding the cross-talk between diabetes and breast cancer

Epidemiological evidence shows that individuals with diabetes have a significantly higher likelihood of developing multiple types of cancers, especially the breast cancer (Larsson, Mantzoros & Wolk, 2007). The mechanisms driving cancer progression in diabetic patients were studied in detail and the significant theories are discussed below.

Recent studies indicate that hyperinsulinemia or administration of synthetic insulin in diabetes may enhance growth factor signaling and increase glucose uptake, leading to tumor growth progression. Hyperglycemia, another characterizing feature of diabetes, may also contribute to enhanced cancer risk (Ryu, Park & Scherer, 2014). Increased glucose uptake is considered as one of the hallmarks of cancer and all types of cancer cells exhibit metabolic rewiring from oxidative phosphorylation to aerobic glycosylation. This altered energy metabolism involves different changes including increased glucose uptake. Moreover, insulin serves as the spark to initiate cancer development at early stages when self-sufficiency of growth factors has not yet been established (illustration HG). Additionally, abnormal levels of O-GlcNAc in cancer cells may contribute to the deregulated posttranslational control of protein function linked to oncogenic phenotypes, for example, O-GlcNAcylation of certain tumor-associated proteins including c-Myc, Ras, beta-catenin, FoxM1, Akt and others to modulate their stability, localization and functions (De Queiroz, Carvalho & Dias, 2014).

## Results and Discussion

**Figure 3:**
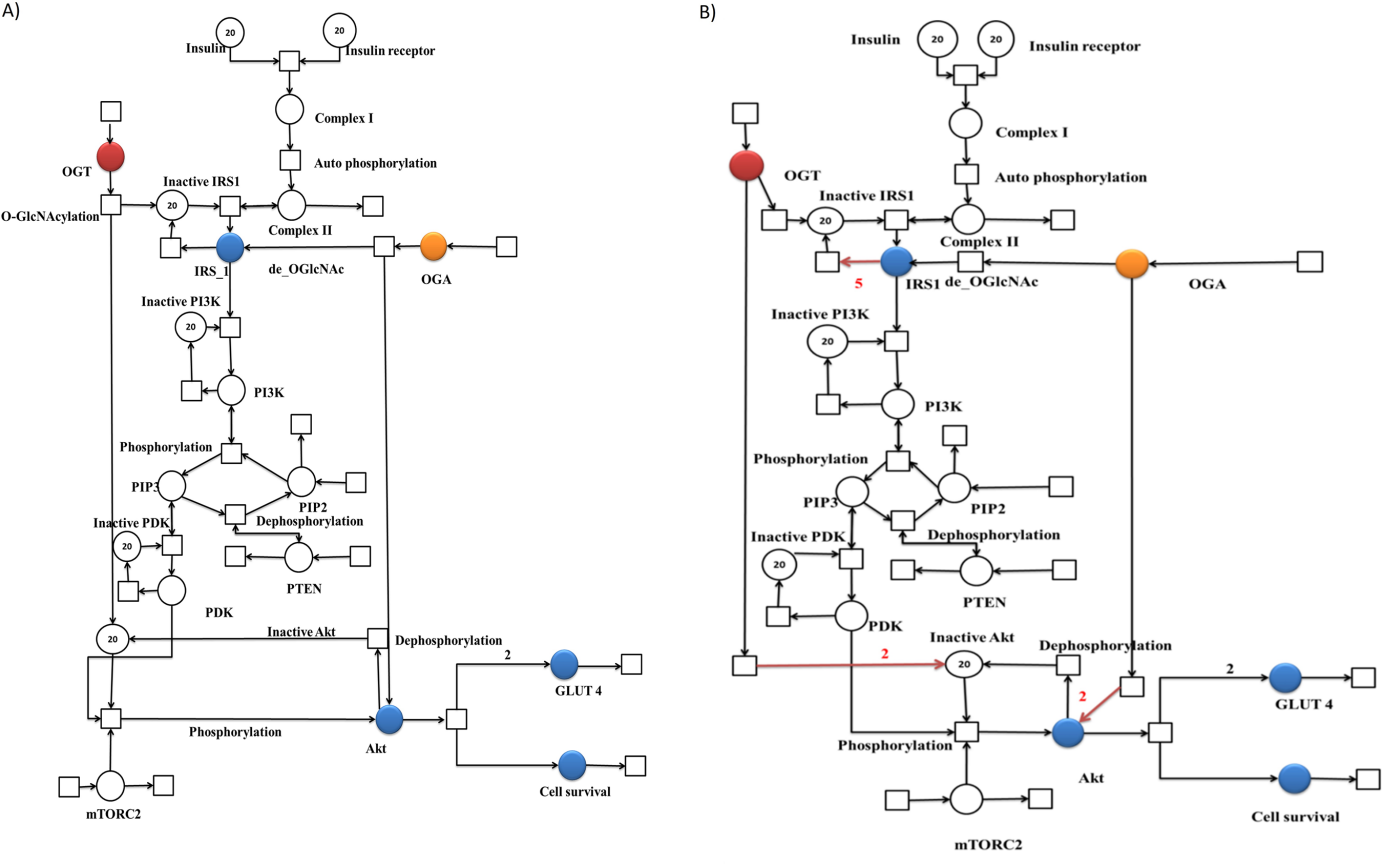
Illustration of the PN models representing altered PI3K/Akt pathway during normal and insulin resistance. A standard place is illustrated as a circle representing proteins involved in the pathway. A continuous transition is depicted as a sqaure representing cellular processes including phosphorylation and dephosphorylation. A directed arc connects a place with a transition and vice versa. Blue places represent important proteins of PI3K pathway selected for this study in particular. (A) PN model of normal PI3K/Akt pathway. Blue places represent important proteins of PI3K/Akt pathway selected for this study in particular. Other colored places include (Red = OGT and Orange = OGA). (B) PN model of altered PI3K/Akt pathway. Blue places represent important proteins of PI3K pathway selected for this study in particular. Red arcs represent the changes in PN model as compared to normal signaling pathway, that is, increased deactivation of IRS-1 and Akt.

The normal PI3K/Akt signaling pathway described in Fig. 1 was modeled using PNs. During model generation, we assumed that when glucose intake occurs, insulin is released into the bloodstream. Meanwhile, insulin receptors are readily available on the cell membrane to initiate the PI3K/Akt pathway via auto-phosphorylation of the receptors and are referred as complex 1 in the proposed models (Fig. 3) (Djiogue et al., 2013; Slawson, Copeland & Hart, 2010).Our base model shows that as soon as the insulin receptor is activated, it recruits and activates the adaptor protein called IRS-1 via phosphorylation. Activated IRS protein displays binding sites for a variety of proteins for further signal transduction. Among them, a major player in insulin function is a PI3K protein that further leads to Akt activation. Activated Akt has numerous functions, among which promoting cell survival through inhibition of pro-apoptotic proteins and regulation of glucose metabolism have been the focus of this study. The complete pathway under normal insulin signaling is shown in Fig. 3A. According to “translocation hypothesis” glucose transporter proteins are present within the cell in the latent state. Upon activation, the number of glucose transporters on the cell membrane is increased and the rate of glucose uptake by the cell is enhanced (Tanti et al., 1997).

### Effect of OGT hyper-activation on insulin resistance

Generally, the concentration of intracellular protein O-GlcNAc modification varies with extracellular glucose concentration. The pathway was formulated and verified for the insulin resistant adipocyte based on the study conducted by Yang et al. (2008), which showed that the approaches used to increase O-GlcNAcylation (adding OGA inhibitor and increasing glucose concentration) inhibited insulin-stimulated phosphorylation of Akt in 3T3-L1 adipocytes (Yang et al., 2008). In Fig. 3B the PN model demonstrates that in an insulin resistant state, OGT function is perturbed, leading to an enhanced inactivation of IRS-1 and Akt. Moreover, this inactivation reduces glucose uptake due to a reduction in the GLUT4 expression on the cell surface, hence decreasing the cell survival rate.

### Comparison of system behavior through model simulations during insulin sensitive and resistant conditions

**Figure 4:**
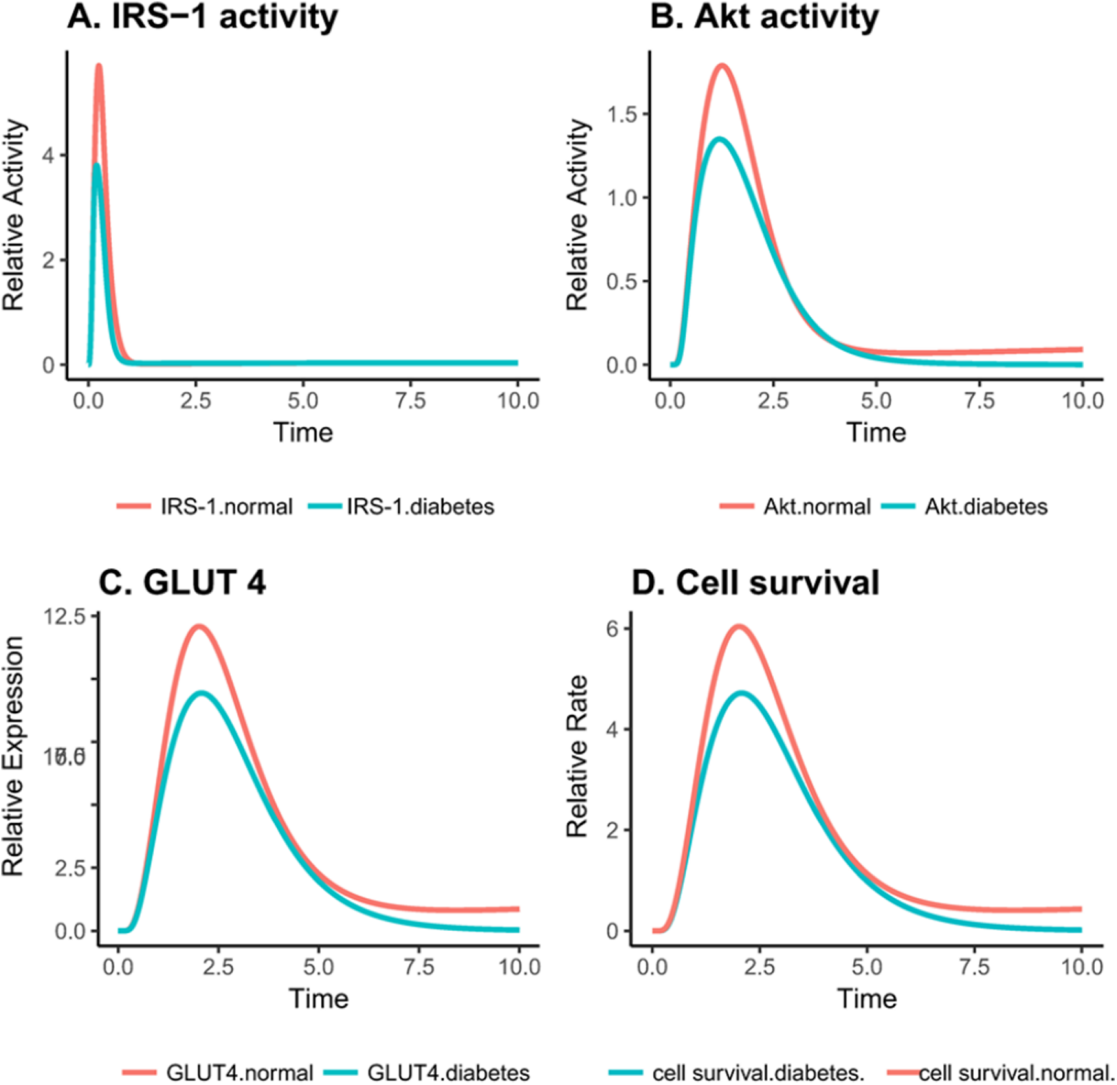
Relative changes in the activity levels of entities in the altered PI3K pathway in insulin-resistant adipocyte. (A) Reduced activation of IRS1 after insulin stimulation. (B) Decreased activity of Akt after insulin stimulation (C) Reduced expression of GLUT4 on the cell surface after insulin stimulation. (D) Decreased cell survival after insulin stimulation.

The complete PI3K/Akt pathway was simulated to study the system as a whole (File S1). Using these simulations, we studied how the proteins behave upon insulin stimulation. It was observed that as soon as insulin binds to insulin receptor there was an increase in activation of downstream proteins. The insulin receptor stayed constant while it initiated the downstream processes by activation of signaling protein, that is, IRS-1. The overall simulation of the altered PI3K/Akt pathway model (File S1) was also analyzed and compared. The comparison revealed that the curves depicting inactive proteins such as inactive IRS-1, PI3K, PDK and Akt show a marked increase with time, representing that with the passage of time inactivity of downstream proteins is increasing and the cell switches from an insulin responsive-cell toward an insulin-resistant phenotype. Interestingly, the dynamics of IRS-1, PI3K and phosphoinositide-dependent kinase 1 (PDK1) were depicted by an increase in their activity as insulin was consumed. These proteins play a key role in insulin signaling pathway thus the individual and collective roles of IRS-1 and Akt and their correlation to OGT protein is analyzed separately (Fig. 4). OGT overexpression lowers the activation of IRS-1 and Akt, therefore, inhibiting insulin signaling in insulin resistance. Moreover, the difference in GLUT-4 expression and cell survival rate, respectively, was also focused during normal and aberrant insulin signaling and is explained below in detail.

As reported by earlier studies, exposing ells to various glucose concentrations does not affect the global concentration of O-GlcNAc but rather the change is protein specific (Yang et al., 2008). It can be seen in Figs. 4A–4C that important proteins such as IRS-1, PI3K, Akt and GLUT-4 show a drop in the activity due to increased O-GlcNAcylation, confirming that insulin signaling is dampened.

### Downregulation of Akt and IRS-1 activity via O-GlcNAcylation during insulin resistance

**Figure 5:**
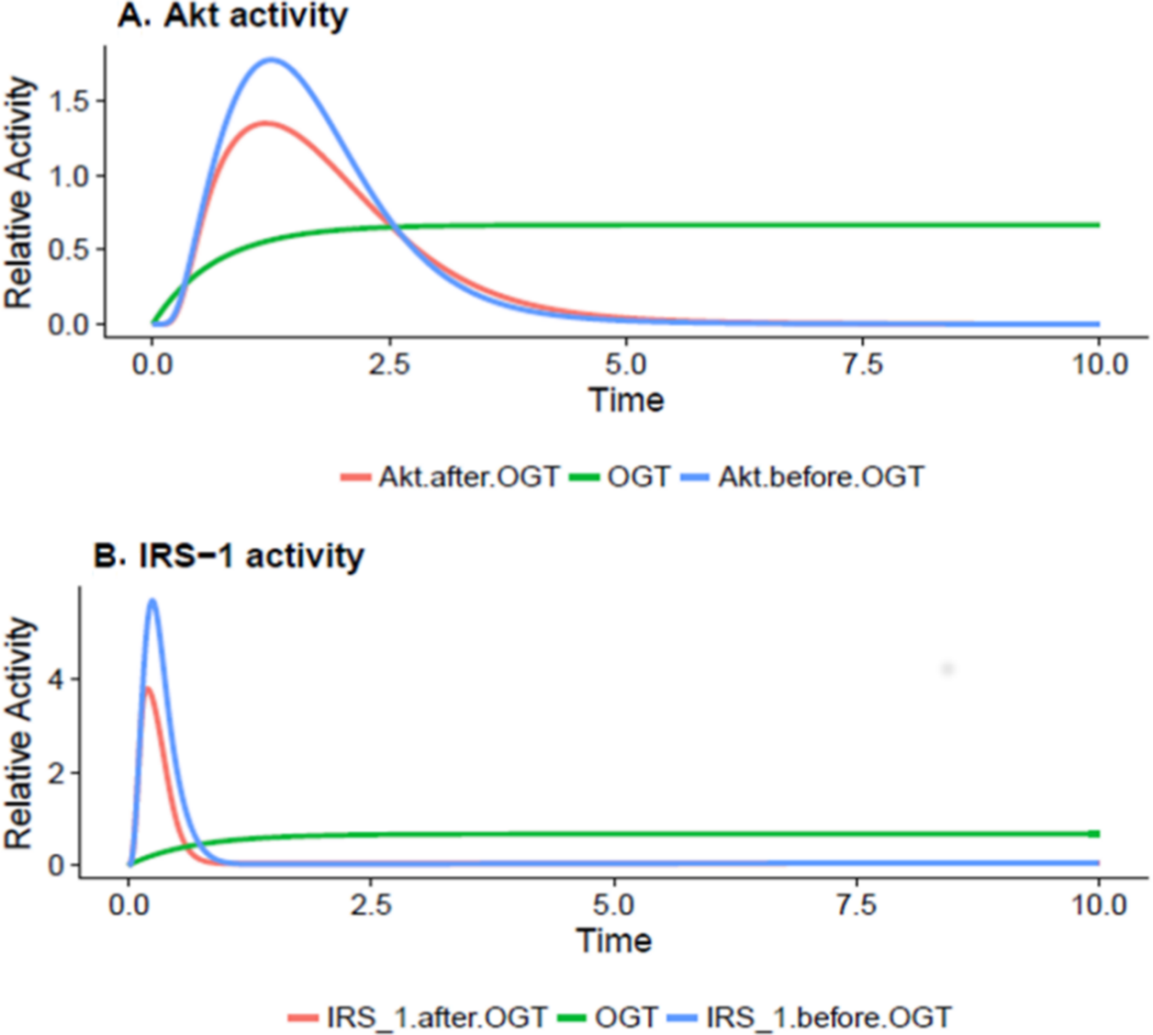
OGT reduces Akt and IRS-OGT reduces Akt and IRS-1 activation via O-GlcNAcylation in insulin-resistant cells. (A) Akt inhibition by OGT in adipocytes. The *x*-axis represents time units while the *y*-axis represents the relative Akt activity. The blue line shows Akt activity before OGT and the red line shows the Akt activity after OGT expression. According to the plot, Akt activity shows a sudden decrease as OGT activity increases (B) Decrease in IRS-1 activity due to O-GlcNAcylation in adipocytes. The *x*-axis shows time units while the *y*-axis shows the relative IRS-1 activity. The blue line represents IRS-1 activity before OGT and the red line presents IRS-1 activity after OGT. The graph explains that IRS-1 activity is significantly reduced as OGT activity increases.

O-GlcNAc transferase carries out regulation of insulin signaling through modifying proteins and altering their activity. In later phase as the adipocyte becomes insulin resistant due to increasing in OGT expression, the values of IRS-1 and Akt drop significantly as increased O-GlcNAcylation inhibits the activation of these proteins. IRS-1 and Akt serve as crucial control points for regulation of insulin-mediated pathway. The reduced activity of IRS-1 and Akt is correlated with inhibition of the PI3K pathway. Figures 5A and 5B show that, during insulin resistance, Akt and IRS-1 activity is significantly reduced via increased O-GlcNAcylation.

Our method computed the activity levels as abstract measures in which the changes over the passage of time depicted changes in active protein concentrations. Therefore, our model was verified as we were able to successfully achieve similar system behavior relative to experimental data (details in Table 1). Henceforth, various biological insights can be driven through extending this model and this signaling pathway can be better understood.

**Table 1:** Summary of the observations as reported by experimental studies and their comparison with our simulations.

Findings					
Observations	Experimental		Model simulation		Citations
	Diabetes	Breast cancer	Diabetes	Breast cancer	
Effect of OGT on IRS-1 Activity	↓	**-**	↓	**-**	Whelan et al. (2010)
Effect of OGT on Akt activity	↓	↑	↓	↑	Whelan et al. (2010), Ahmad, Singh & Glazer (1999)
GLUT-4 expression	↓	↑	↓	↑	Postic et al. (1993)
Effect of OGT on cell survival	↓	↑	↓	↑	Caldwell et al. (2010), Postic et al. (1993)

**Note:**

Symbols represent changes in expression levels of observed proteins in the PI3K/Akt pathway. ↑ represents the up-regulation while ↓ represents down-regulation of the entities/proteins. **-** represents non-availability of data.

### Effect of hyperglycemic condition on the expression of OGT in invasive breast cancer

Hyperglycemia contributes to malignant cancer cell phenotypes (Ryu, Park & Scherer, 2014). There is increasing evidence suggesting that there is a link between cancer and diabetes. Regardless of other shared metabolic factors, hyperglycemia, the most typical characteristic of diabetes, may be one of the explanations for the prevalence of cancer incidence in patients with diabetes. Research shows that hyperglycemia may contribute to an enhanced proliferation ability, apoptosis inhibition, metastasis, perineural invasion, chemotherapy resistance and chemotherapy intolerance (Duan et al., 2014).

Glucose is specifically required to meet the metabolic demands of the fast proliferation of cancer cells. It is known that glucose is a primary driving force for the growth of tumor cells (Beckner et al., 1990). The significant role of hyperglycemia in cancer proliferation is clearly understood. Hyperglycemia is often accompanied by hyperinsulinemia in people with diabetes. Moreover, recent studies showed that insulin promotes cancer progression by enhancing metabolic capacities of cancer cells (Iqbal et al., 2013). Naturally, OGT is highly abundant in β cells in the islet (Hanover et al., 1999) and O-GlcNAc levels in β cells are sensitive to glucose (Liu et al., 2000), implying that O-GlcNAc may function as a glucose sensor to regulate insulin secretion. O-GlcNAc regulates insulin signaling in response to glucose flux, thus hyperglycemic condition leads to elevated O-GlcNAc modifications in response to increased flux through the HBP.

**Figure 6:**
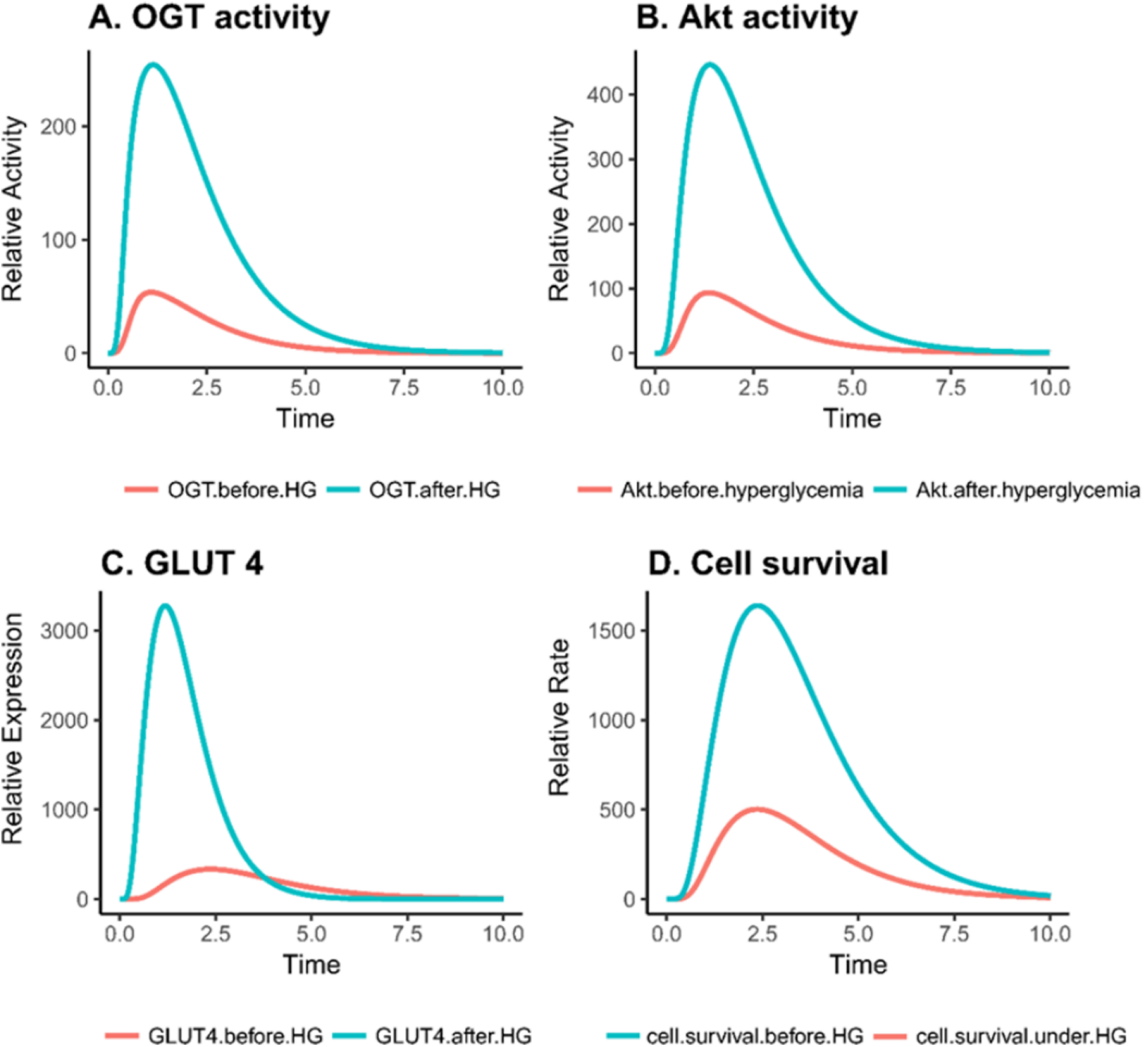
Relative changes in the activity levels of entities in breast cancer under hyperglycemia. (A) Increased activity of OGT in invasive breast cancer. (B) Hyper activation of Akt in breast cancer cell under hyperglycemic condition. (C) Increase in GLUT4 expression due to increased PI3K activity. (D) Increase in cell survival due to increased activation of PI3K activity.

Based on PN simulation, Figs. 6A–6D depict the exponential increase in protein expression and cell survival under hyperglycemic condition. As hyperglycemia induces increased OGT expression, it results in hyper-activation of PI3K/Akt pathway via O-GlcNAcylation in breast cancer. Moreover, there is a significant increase in the GLUT 4 expression and cell survival rate of breast cancer cell as shown in Figs. 6C–6D. It was observed that there was an approximately fourfold increase in OGT and a 10-fold increase in cell proliferation (Table 2). This verifies that our model is sound and predicts these behaviors correctly.

**Table 2:** Summary of the observations derived from simulated results.

Effect of:	OGT (relative units)	Cell proliferation (relative units)
Hyperglycemia	>4-fold	>10-fold
shRNA	<0.2-fold	<0.3-fold
BZX	<7-fold	<14-fold
Metformin	No change	<5-fold
BZX + Metformin	<7-fold	<20-fold

### Comparison of differential role of OGT during insulin resistant condition and invasive breast cancer

The designed PN model represents how increased feedback inhibition of the PI3K/Akt pathway due to dysregulated O-GlcNacylation of Akt in adipocytes causes the cell to become resistant to insulin. This results in reduced insulin utilization and causes hyperinsulinaemia. Consequently, PI3K/Akt pathway stimulation is decreased. These cells then reduce the expression of GLUT 4 receptors leading to reduced glucose uptake. In turn, hyperglycemic condition prevails in the blood stream.

**Figure 7:**
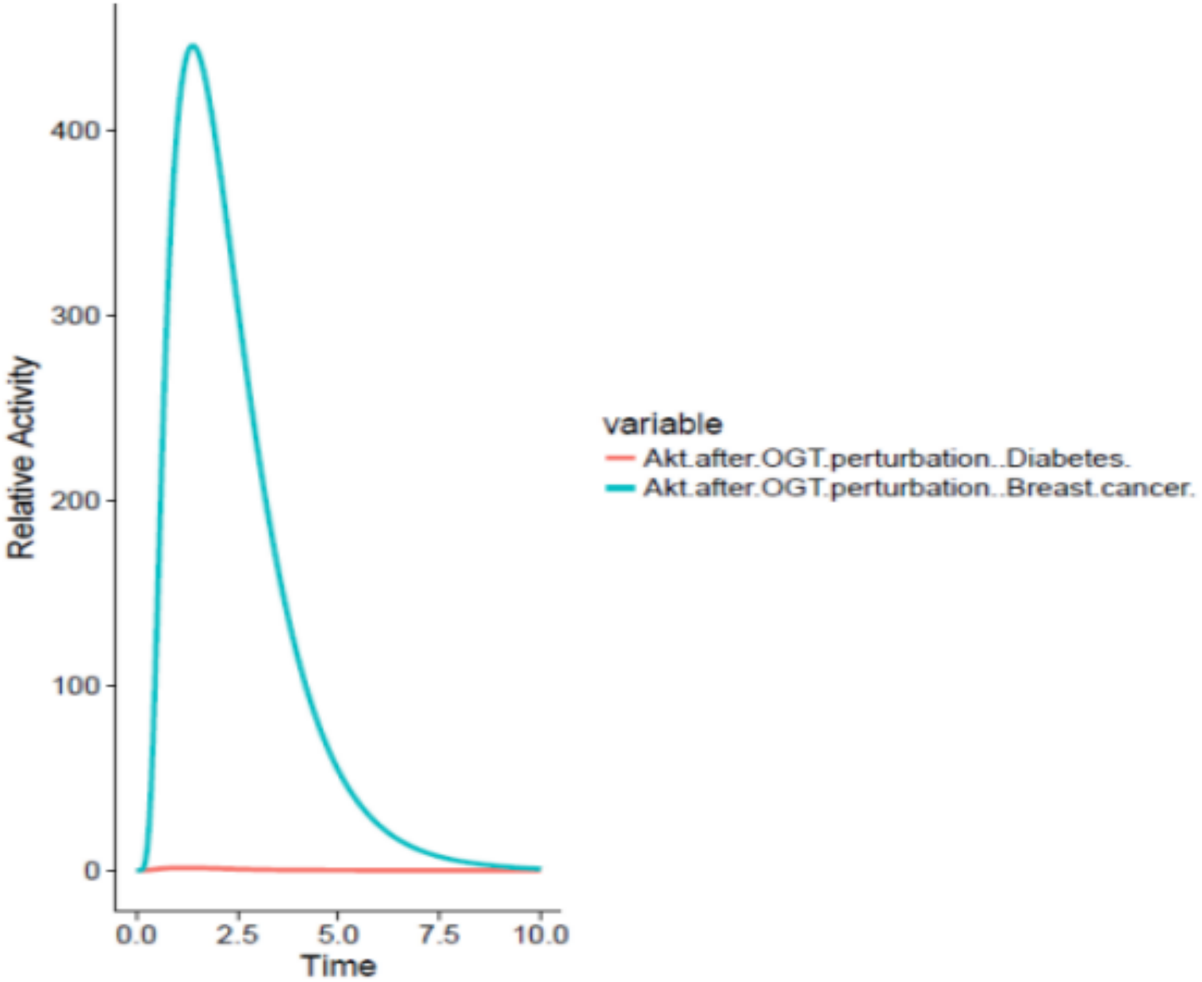
Differential role of OGT. The *x*-axis shows time units while *y*-axis shows the relative activity of Akt. The red line represents Akt activity after OGT hyperactivity in diabetes whereas the blue line represents Akt activity after OGT hyperactivity in breast cancer. The graph shows that there is an exponential increase in Akt activity as aberrant O-GlcNacylation enhances Akt in the breast cancer as compared to its activity in diabetes.

On the other hand, OGT resulted in hyper activation of the PI3K/Akt pathway in the breast cancer cell by increased O-GlcNacylation of Akt leading to increased glucose uptake by the cancer cell through GLUT4 expression as well as increased cell survival. The graph comparing the Akt activation in adipocytes and breast cancer cell has been shown in Fig. 7.

Since hyperactivity of OGT induces cancer cell proliferation and survival, thus inhibition of OGT at the appropriate level would be beneficial in breast cancer patients having diabetes. In order to study the effects of such kind of inhibition, we designed perturbation experiments to inhibit OGT through specific RNA and protein inhibitors and are discussed as separate cases below.

### CASE 1: effect of shRNA interference on OGT expression during invasive breast cancer

Short hairpin or small hairpin RNA (shRNA) is an artificial RNA molecule with a hairpin structure that is used to carry out RNA interference through targeting gene expression. shRNA expression is achieved in cells through plasmid delivery or vectors. Low turnover and decreased degradation rate make shRNA, an effective mediator of RNA interference. The data of shRNA used for this experiment was taken from a study conducted by Caldwell et al. (2010).

**Figure 8:**
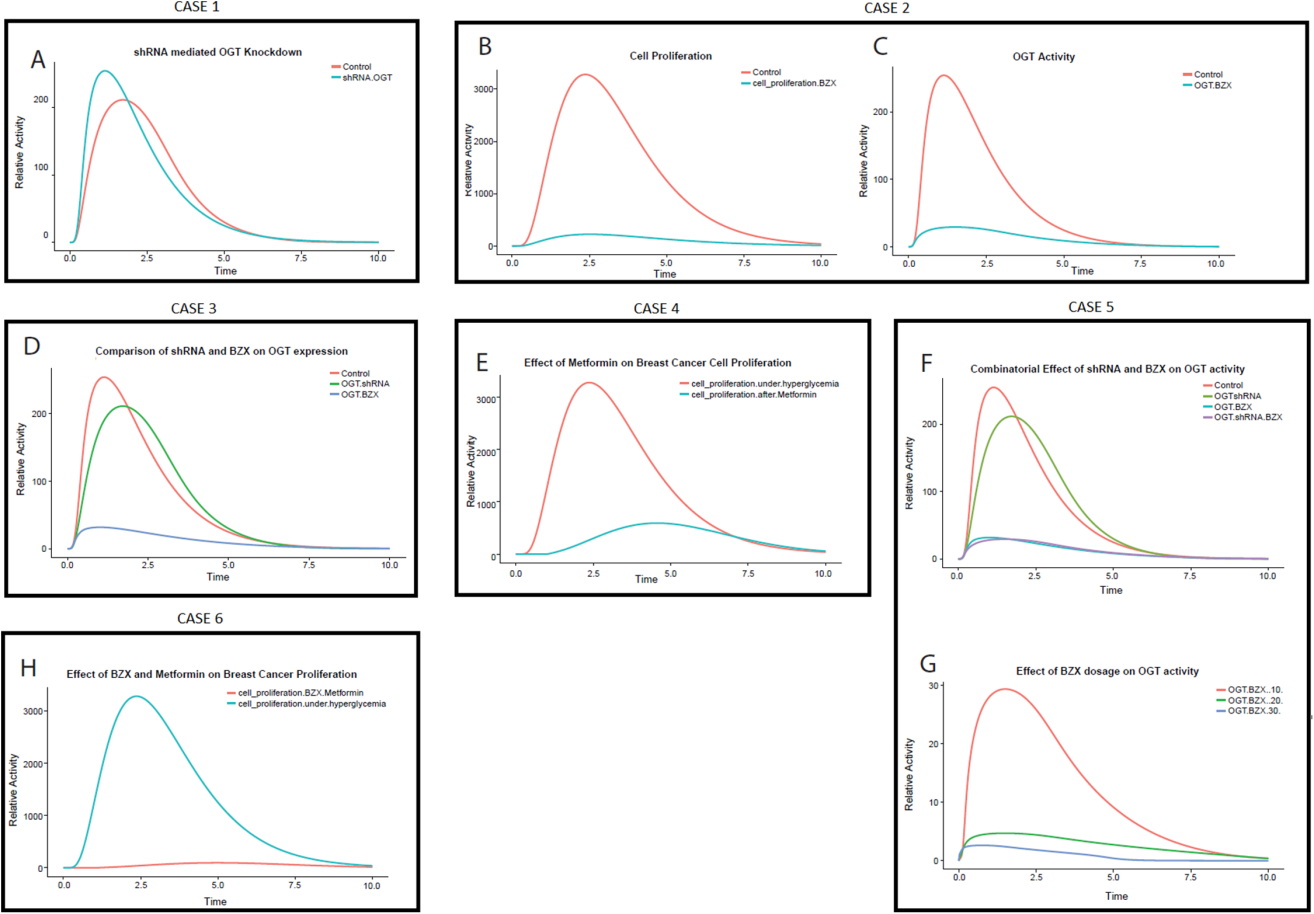
Perturbation experiments (cases) designed to inhibit OGT through specific RNA and protein inhibitors. (A) Relative effect of various cases OGT inhibition by shRNA drops the OGT activity in breast cancer cell The *x*-axis shows time units while the *y*-axis shows relative OGT activity. The blue line represents OGT activity under hyperglycemia whereas the red line represents OGT activity after shRNA intervention. (B) Effect of OGT inhibitor BZX on breast cancer cell proliferation. The *x*-axis shows the time units while the *y*-axis represents the relative rate of breast cancer cell proliferation. Red line represents cell proliferation under hyperglycemia whereas blue line represents cell proliferation after BZX intervention. The graph shows a drastic reduction in breast cancer cell proliferation by BZX. (C) OGT activity reduces significantly by BZX. The figure shows that OGT levels drop drastically due to BZX inhibition. The red line shows OGT activity under hyperglycemia (control) whereas blue line shows OGT activity after BZX. (D) Relative efficacy of shRNA and BZX on OGT activity. (E) Effect of Metformin on breast cancer cell proliferation. The *x*-axis shows the time units while the *y*-axis represents the relative rate of breast cancer cell proliferation. The red line represents cell proliferation under hyperglycemia whereas the blue line represents cell proliferation after Metformin. (F) Combinatorial effect of shRNA and BZX on OGT activity. The *x*-axis shows the time units while the *y*-axis represents the relative activity of OGT. (G) Increasing BZX dosage reduces OGT activity considerably. The *x*-axis shows the time units while the *y*-axis represents the relative activity of OGT. When BZX was increased to 30 units, OGT activity decreased completely, much earlier than that at BZX at 20 and 10. (H) Effect of Metformin on breast cancer cell proliferation.

The PN model (File S2) is designed to study the effect of shRNA interference on OGT in breast cancer. The model describes that the addition of shRNA against OGT RNA binds and degrades the OGT mRNA. The simulation run was performed which is shown in Fig. 8A. It is observed that OGT activity decreases with time. Approximately a 0.2-fold decrease in OGT activity is observed (Table 2). This can be significant in reducing the active load of OGT in the cell.

### CASE 2: effect of BZX on OGT expression during invasive breast cancer

Over the years, high-throughput screening methods against a huge library of drug-like composites have produced a number of OGT inhibitors. Application of this screening method has detected a compound called 4-methoxyphenyl 6- acetyl-2-oxobenzo[d]oxazole-3(2H)-carboxylate also denoted as BZX that competes with the binding of sugar nucleotide. It is a suicide inhibitor that cross-links the active site of OGT (Lys842 and Cys917) through a double-displacement mechanism (Ostrowski & Van Aalten, 2013).

We chose BZX, as it has successfully been used against breast cancer in vitro, promoting anti-invasion and antigrowth effects as it modulated FoxM1 transcription (Caldwell et al., 2010). Recent studies revealed that the use of BZX against OGT downregulated the expression of genes that are linked to cell cycle regulation, DNA replication and also destabilized c-Myc (a known oncogene) in human prostate cell lines (Heiner et al., 2012). This compound has further been used to study the significance of O-GlcNAcylation through modulation of Rho kinase pathway in vascular contractile response (Lima et al., 2010).

Using data generated through previous research, we applied BZX to our invasive breast cancer model to study its effect on OGT activity and breast cancer cell proliferation (File S3). Interestingly, our results show that OGT inhibition via BZX decreased cell proliferation significantly (Figs. 8B and 8C), that is, by 14-fold as given in Table 2. Moreover, BZX inhibition of OGT (Figs. 8B and 8C) was more effective than shRNA interference as it reduced OGT activity by sevenfolds. This finding confirms the role of OGT in regulating cell growth and invasion as studied in breast cancer cells by Caldwell et al. (2010).

### CASE 3: relative efficacy of shRNA and BZX during invasive breast cancer

Comparison of OGT mRNA interference and OGT inhibition was done in order to understand the relative effect of shRNA and BZX. Figure 8D describes the difference between the two strategies. Red line represents OGT activity under hyperglycemia (control), Green line represents the effect of shRNA on OGT activity and the Blue line represents the effect of BZX on OGT activity. Figure 8D shows that the inhibition by BZX is more effective as compared to shRNA inhibition. Based on our model, OGT inhibition by BZX produced better results compared to shRNA interference as OGT activity dropped significantly through BZX inhibition.

### CASE 4: effect of Metformin on invasive breast cancer cell proliferation

A well-known anti-diabetic drug, Metformin has been used to study its anti-tumor activity in a variety of cancers, including breast cancer (Li, 2011). This drug is used very commonly and has well-established safety profiles (Barnett et al., 2006). Using data from the literature, we carried out inhibition of breast cancer cell proliferation via Metformin. The PN model (File S4) that was subjected to Metformin intervention whereas Fig. 8E shows the simulation for this intervention.

The graph in Fig. 8E shows that with the passage of time, Metformin reduces breast cancer cell proliferation up to approximately fivefolds. The uncontrolled cell proliferation at the start is due to an immense increase in glucose flux leading to HBP. However, the rate of cell proliferation decreases as Metformin is applied showing indirect inhibition of cell proliferation over time.

### CASE 5: combination therapy (shRNA + BZX)

A therapy consisting of more than one modality or medication to treat a single disease is known as a combination therapy. To analyze the combinatorial effect of OGT inhibition through shRNA and BZX, PN model was formulated (File S5) and the effect of shRNA and BZX in combination on OGT activity is represented in Figs. 8F and 8G. Figure 8F shows that the inhibition by a combination shRNA and BZX did not show a substantial difference; therefore, we tested increase in dosage of BZX with time. Increasing BZX dosage by 10 units with time, we observed a visible decline in OGT activity. We tested three values by giving BZX a token of 10, 20 and 30. The graph in Fig. 8G explains the effect of increasing BZX dosage on OGT activity.

### CASE 6: combination therapy (BZX + Metformin)

To check the efficacy of combination therapy including OGT inhibitor BZX and Metformin on breast cancer cell proliferation another PN was designed (File S6).

We used this combination to check whether OGT inhibition and blocking cell proliferation will impair breast cancer progression further. Also, the interaction of Metformin with OGT has not yet been reported, therefore this combination targets two separate processes. Based on our calculations, up to 20-fold decrease in cell proliferation was observed (Table 2). Fig. 8H depicts that breast cancer cell proliferation becomes almost negligible through this drug combination. This strategy can prove to be very beneficial as it not only decreases breast cancer cell proliferation but also inhibits OGT that aggravates cancer by modifying central players in cancer progression such as Akt.

The observations of perturbation experiments in terms of fold changes have been summarized in Table 2. The table shows relative fold changes in OGT protein and cell proliferation due to hyperglycemia and the effect of the application of various therapies in breast cancer. The observations predict that the combination therapy with BZX and Metformin gave simultaneously is far more effective than sequential therapy. It can prove to be comparatively better as it not only decreases OGT expression but also reduces breast cancer cell proliferation significantly.

## Conclusion

The PN model verified that alterations in O-GlcNAc signaling affect both insulin resistance and breast cancer. Under the hyperglycemic state, cell survival, growth and proliferation of breast cancer were greatly enhanced. Moreover, the combination therapy for breast cancer patients consisting of anti-diabetic drugs such as Metformin along with OGT inhibitors for example BZX can produce better treatment regimens.

In the future, more OGT inhibitors can be tested using this model to better understand their efficacy for treatment of diabetes and breast cancer. Moreover, the role of O-GlcNAcylation in the onset of diabetes and in diabetic complications should be elucidated further. It is also to be expected that therapeutic agents that target O-GlcNAcylation will become available for the treatment of diabetes and its complications.

Furthermore, numerous studies show a correlation between increased hexosamine biosynthetic flux and insulin resistance; however, the relationship between the two mechanisms has not been established. Although the relationship between HBP and insulin resistance may be quite complex, it clearly deserves further study in concert with its role in the complications of diabetes.

## Supplemental Information

10.7717/peerj.5917/supp-1 Supplemental Information 1 Fig S1. Behavior of PI3K/Akt pathway in adipocytes before and after OGT hyper-activation. **(A)** Normal behavior. The graph shows a collective simulation of 20 entities in the PI3K/Akt pathway under normal condition. The relative activity change in protein levels is measured in response to insulin stimulation of PI3K/Akt pathway. **(B)**. Altered behavior. The graph shows a collective simulation of 20 entities in the PI3K/Akt pathway under altered PI3K/ Akt pathway. The shift in relative activity of proteins levels is represented as the cell becomes insulin resistant. The relative activity change in protein levels is measured in response to insulin stimulation of PI3K/Akt pathway.

10.7717/peerj.5917/supp-2 Supplemental Information 2 Fig S2. Illustration of the PN model representing an intervention in Hexosamine Biosynthetic pathway through shRNA in breast cancer cell under hyperglycemia. A standard place is illustrated as a circle representing proteins involved in the pathway. A continuous transition is depicted as a square representing cellular processes including phosphorylation and dephosphorylation. A directed arc connects a place with a transition and vice versa. Red arcs represent changes in PN as compared to normal PI3K/Akt pathway i.e. OGTmRNA degradation via shRNA, increased cell proliferation, and GLUT-4 expression. Colored places include (Blue = Akt, Light blue = shRNA OGT, Red= OGT and Orange = OGA and Pink= increased glucose molecules).

10.7717/peerj.5917/supp-3 Supplemental Information 3 Fig S3. Illustration of the PN model representing BZX inhibition of OGT. A standard place is illustrated as a circle representing proteins involved in the pathway. A continuous transition is depicted as a square representing cellular processes including phosphorylation and dephosphorylation. A directed arc connects a place with a transition and vice versa. Red arcs represent the action of BZX on OGT. Colored places include (Blue = Akt, Green = BZX, Red= OGT and Orange = OGA).

10.7717/peerj.5917/supp-4 Supplemental Information 4 Fig S4. Illustration of the PN model representing Metformin intervention. A standard place is illustrated as a circle representing proteins involved in the pathway. A continuous transition is depicted as a square representing cellular processes including phosphorylation and dephosphorylation. A directed arc connects a place with a transition and vice versa. Inhibitory arc is represented in red color showing inhibitory effect of Metformin on cell proliferation. Colored places include (Blue = Akt, Purple = Metformin, Red= OGT and Orange = OGA).

10.7717/peerj.5917/supp-5 Supplemental Information 5 Fig S5. Illustration of the PN model representing shRNA intervention. A standard place is illustrated as a circle representing proteins involved in the pathway. A continuous transition is depicted as a square representing cellular processes including phosphorylation and dephosphorylation. A directed arc connects a place with a transition and vice versa. Red arcs represent increase in cell proliferation and GLUT-4 expression under hyperglycemia. Colored places include (Green = BZX, Red= OGT and Orange = OGA, Pink = increased glucose molecules and Light blue = shRNA).

10.7717/peerj.5917/supp-6 Supplemental Information 6 Fig S6. Illustration of the PN model representing BZX and Metformin intervention in breast cancer cell under hyperglycemia. A standard place is illustrated as a circle representing proteins involved in the pathway. A continuous transition is depicted as square representing cellular processes including phosphorylation and dephosphorylation. A directed arc connects a place with a transition and vice versa. Inhibitory arc is represented by an arc with a hollow dot as its head in red color representing inhibition of cell proliferation by Metformin. Colored places include (Blue = Akt, Green = BZX, Red= OGT and Orange = OGA and Purple = Metformin).

10.7717/peerj.5917/supp-7 Supplemental Information 7 Supplementary files with raw data of Petri net models. Supplementary files contain all the raw data and models of the Petri nets. It can be opened in Snoopy tool available at http://www-dssz.informatik.tu-cottbus.de/DSSZ/Software/Snoopy.

## Additional Information and Declarations

### Data Availability

The following information was supplied regarding data availability: The raw data are provided in the Supplemental Files.
