# Supplementary figures and images for: Model-based in silico analysis of the PI3K/Akt pathway: the elucidation of cross-talk between diabetes and breast cancer
Source: PeerJ. 2018 Nov 9;6:e5917. doi: 10.7717/peerj.5917 (PMC6265603; doi:10.7717/peerj.5917)

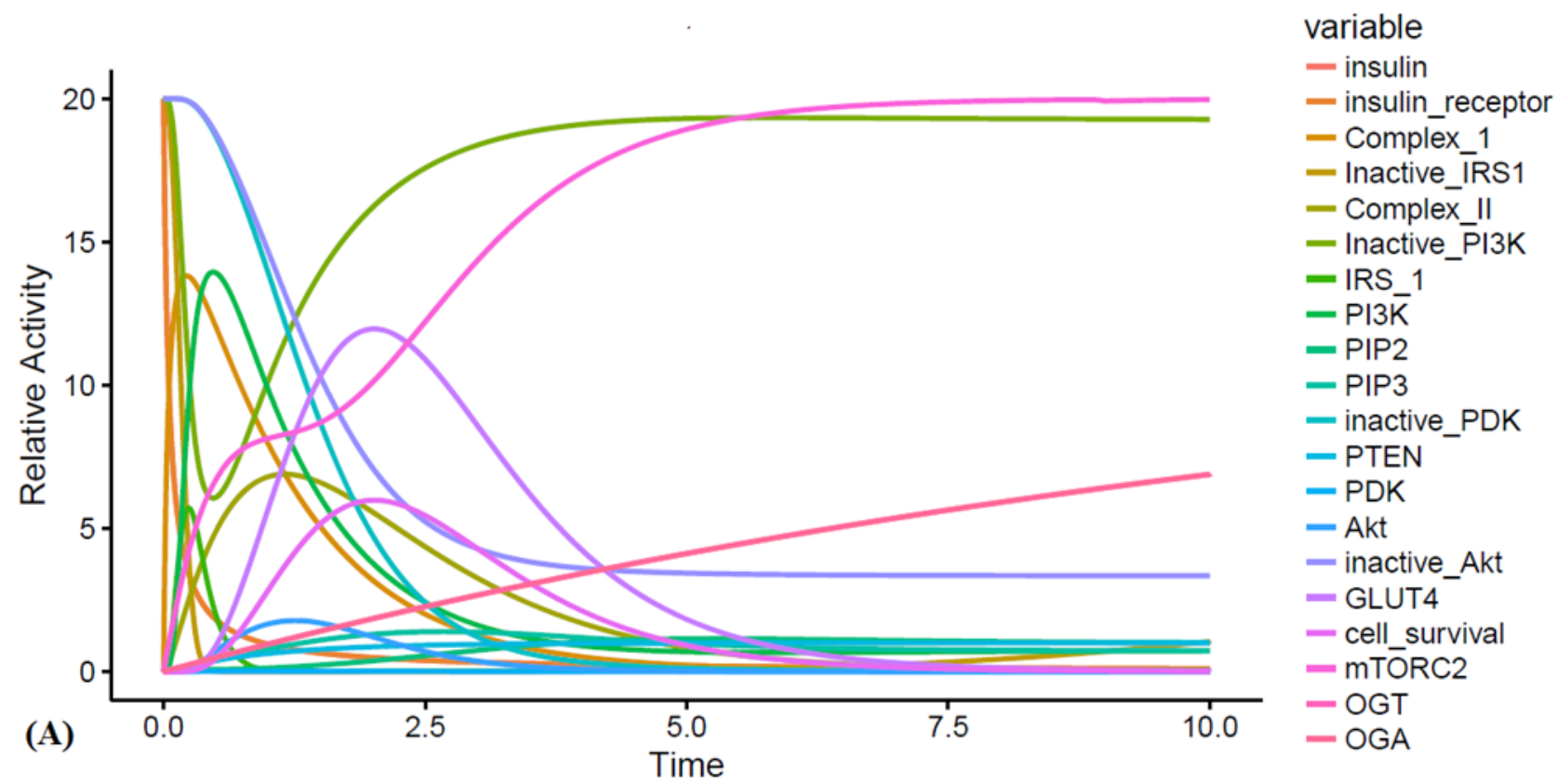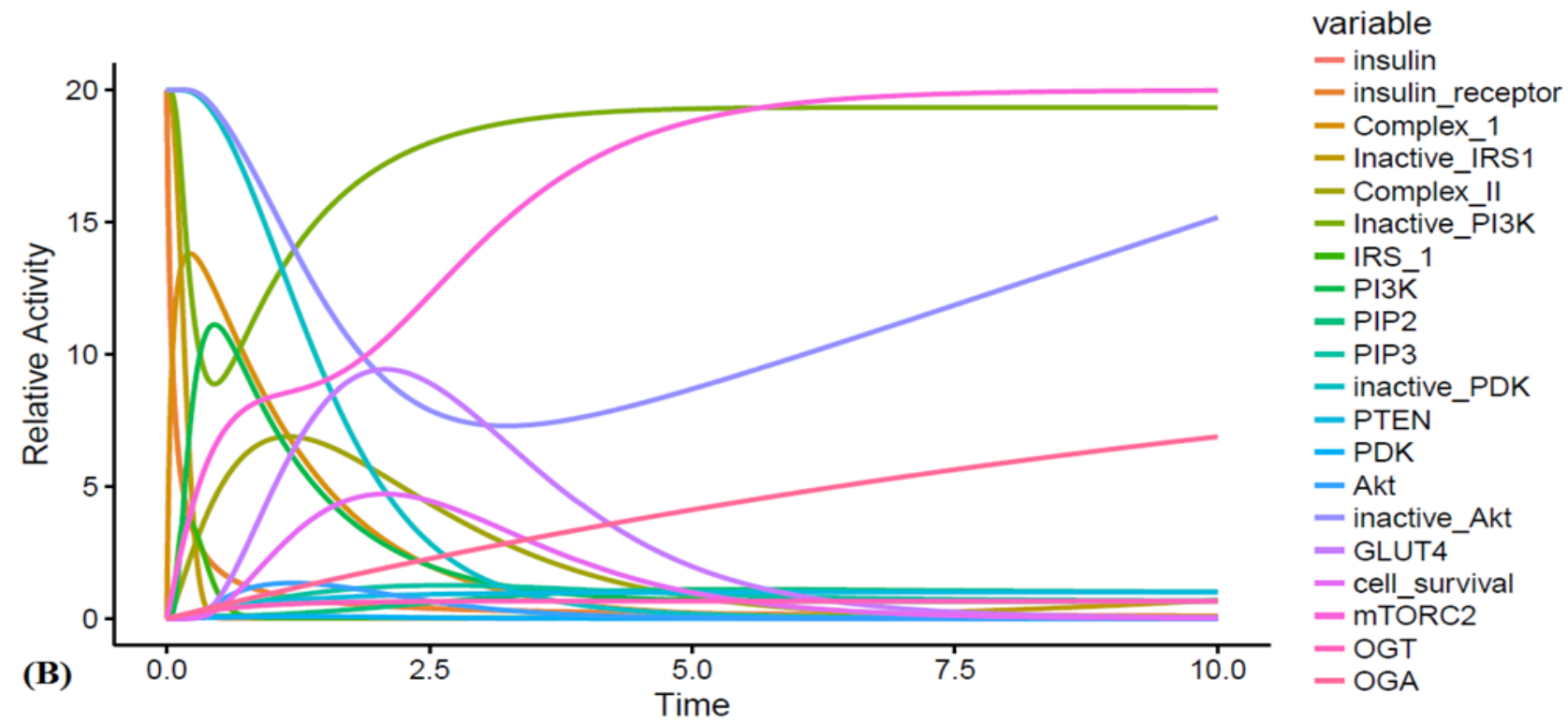

Supplement: Supplemental Information 1 — (A) Normal behavior. The graph shows a collective simulation of 20 entities in the PI3K/Akt pathway under normal condition. The relative activity change in protein levels is measured in response to insulin stimulation of PI3K/Akt pathway. (B). Altered behavior. The graph shows a collective simulation of 20 entities in the PI3K/Akt pathway under altered PI3K/ Akt pathway. The shift in relative activity of proteins levels is represented as the cell becomes insulin resistant. The relative activity change in protein levels is measured in response to insulin stimulation of PI3K/Akt pathway. [file peerj-06-5917-s001.pdf]

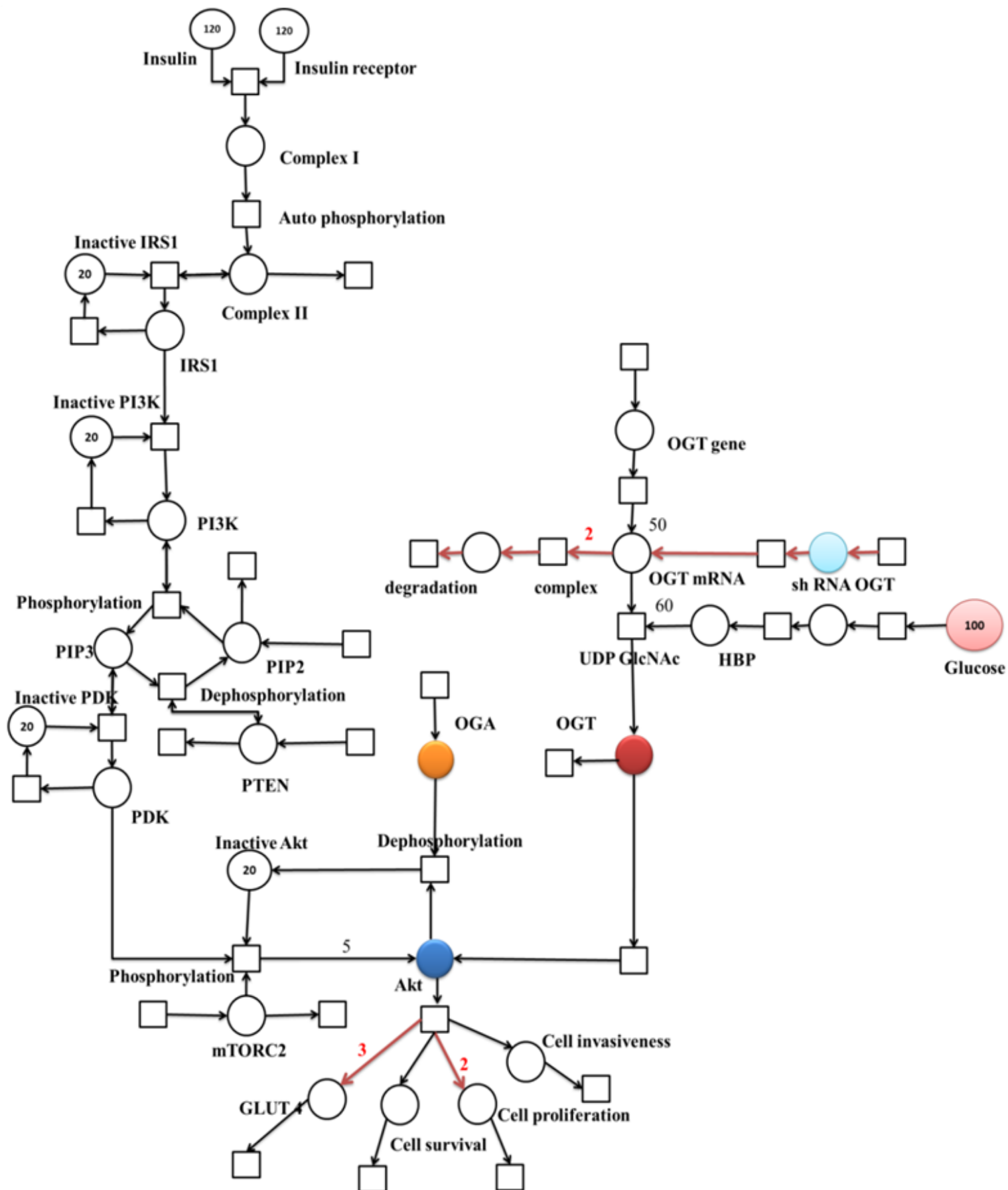

Supplement: Supplemental Information 2 — A standard place is illustrated as a circle representing proteins involved in the pathway. A continuous transition is depicted as a square representing cellular processes including phosphorylation and dephosphorylation. A directed arc connects a place with a transition and vice versa. Red arcs represent changes in PN as compared to normal PI3K/Akt pathway i.e. OGTmRNA degradation via shRNA, increased cell proliferation, and GLUT-4 expression. Colored places include (Blue = Akt, Light blue = shRNA OGT, Red= OGT and Orange = OGA and Pink= increased glucose molecules). [file peerj-06-5917-s002.pdf]

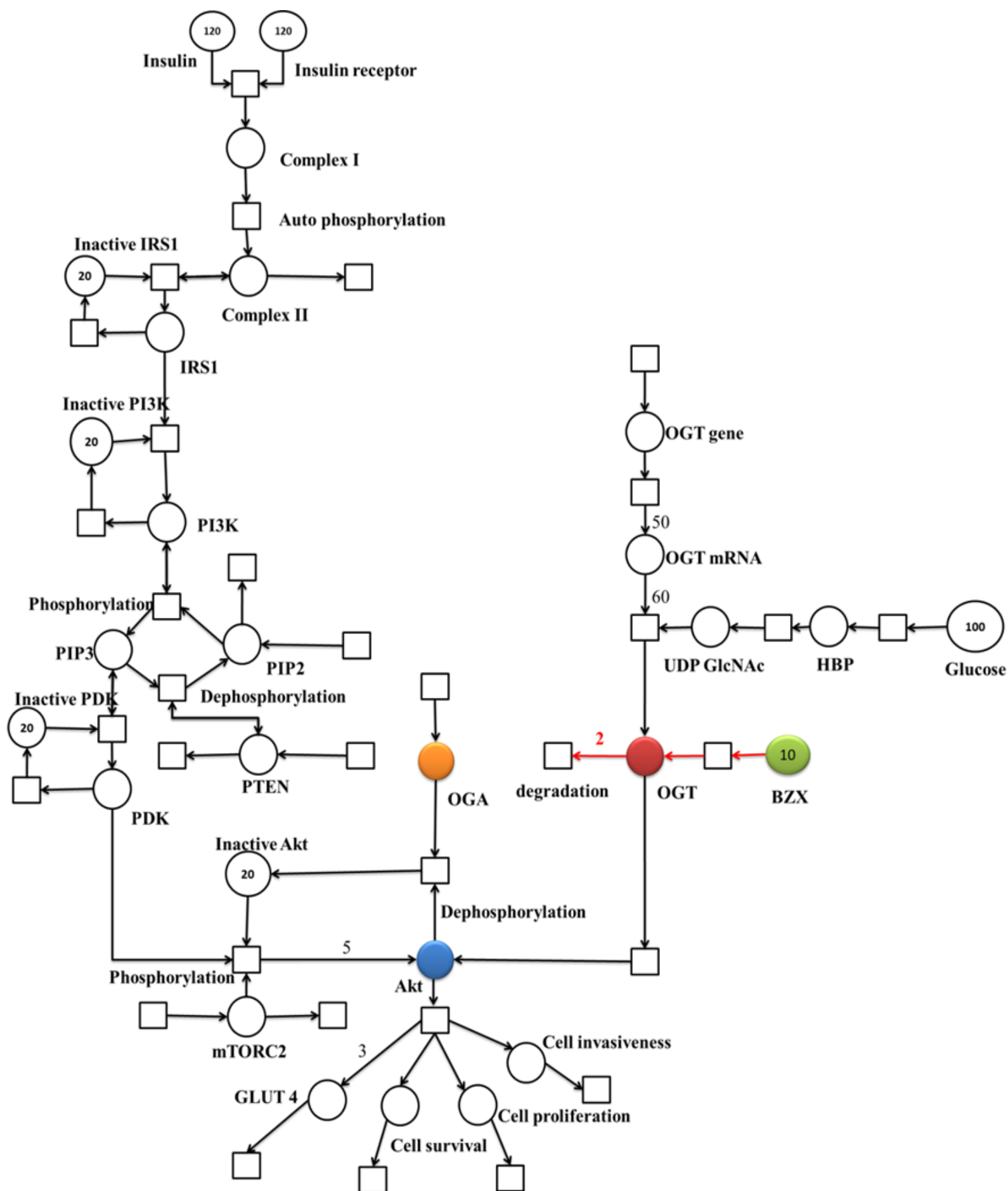

Supplement: Supplemental Information 3 — A standard place is illustrated as a circle representing proteins involved in the pathway. A continuous transition is depicted as a square representing cellular processes including phosphorylation and dephosphorylation. A directed arc connects a place with a transition and vice versa. Red arcs represent the action of BZX on OGT. Colored places include (Blue = Akt, Green = BZX, Red= OGT and Orange = OGA). [file peerj-06-5917-s003.pdf]

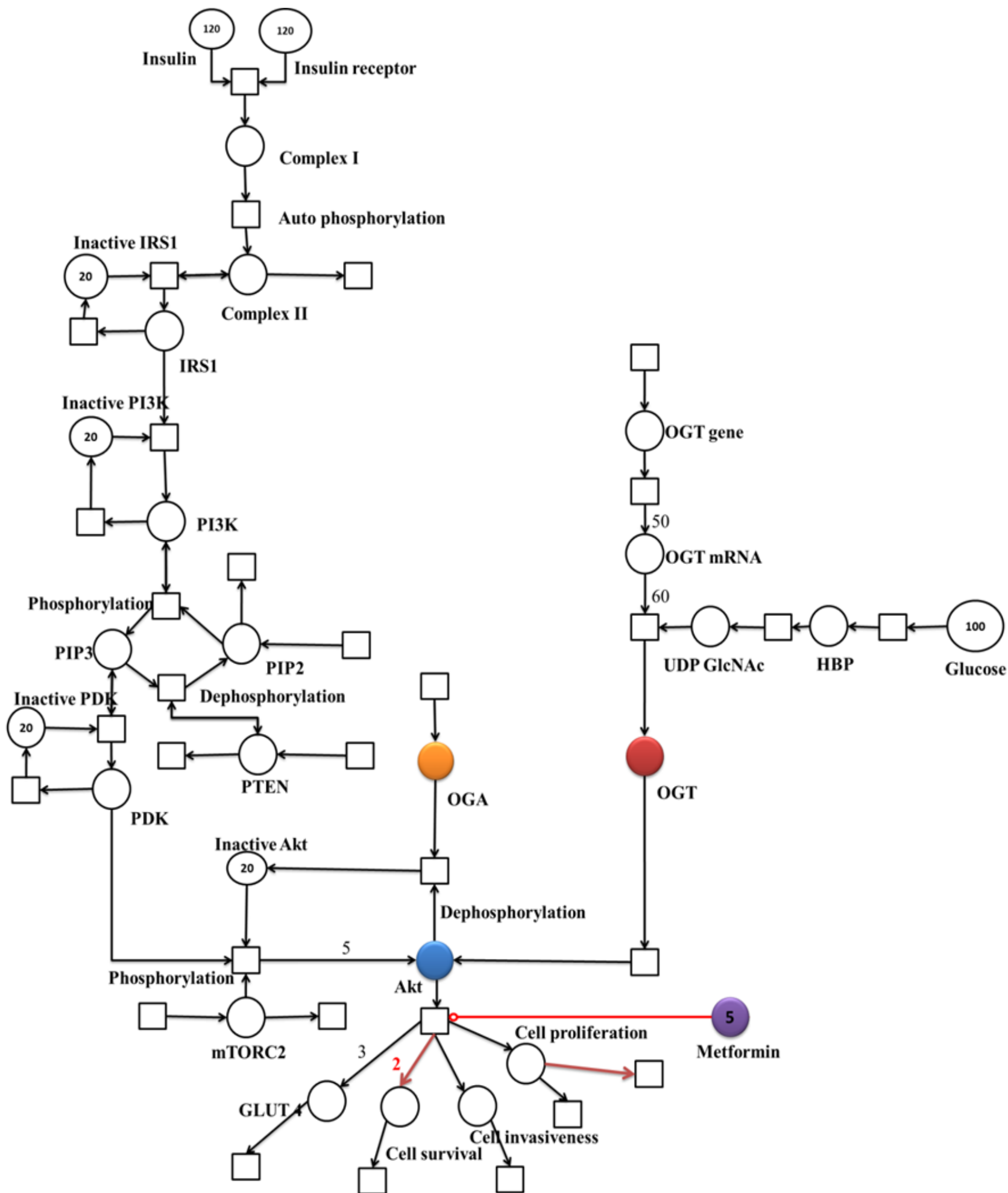

Supplement: Supplemental Information 4 — A standard place is illustrated as a circle representing proteins involved in the pathway. A continuous transition is depicted as a square representing cellular processes including phosphorylation and dephosphorylation. A directed arc connects a place with a transition and vice versa. Inhibitory arc is represented in red color showing inhibitory effect of Metformin on cell proliferation. Colored places include (Blue = Akt, Purple = Metformin, Red= OGT and Orange = OGA). [file peerj-06-5917-s004.pdf]

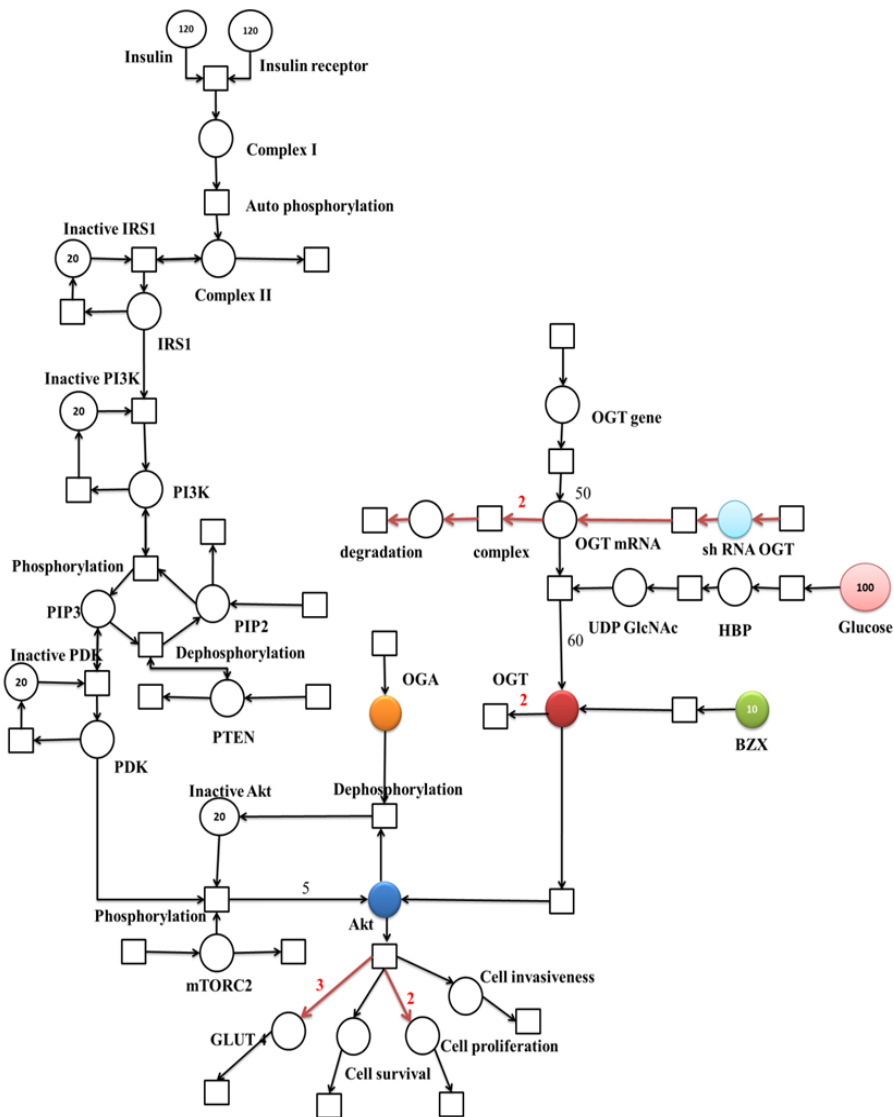

Supplement: Supplemental Information 5 — A standard place is illustrated as a circle representing proteins involved in the pathway. A continuous transition is depicted as a square representing cellular processes including phosphorylation and dephosphorylation. A directed arc connects a place with a transition and vice versa. Red arcs represent increase in cell proliferation and GLUT-4 expression under hyperglycemia. Colored places include (Green = BZX, Red= OGT and Orange = OGA, Pink = increased glucose molecules and Light blue = shRNA). [file peerj-06-5917-s005.pdf]

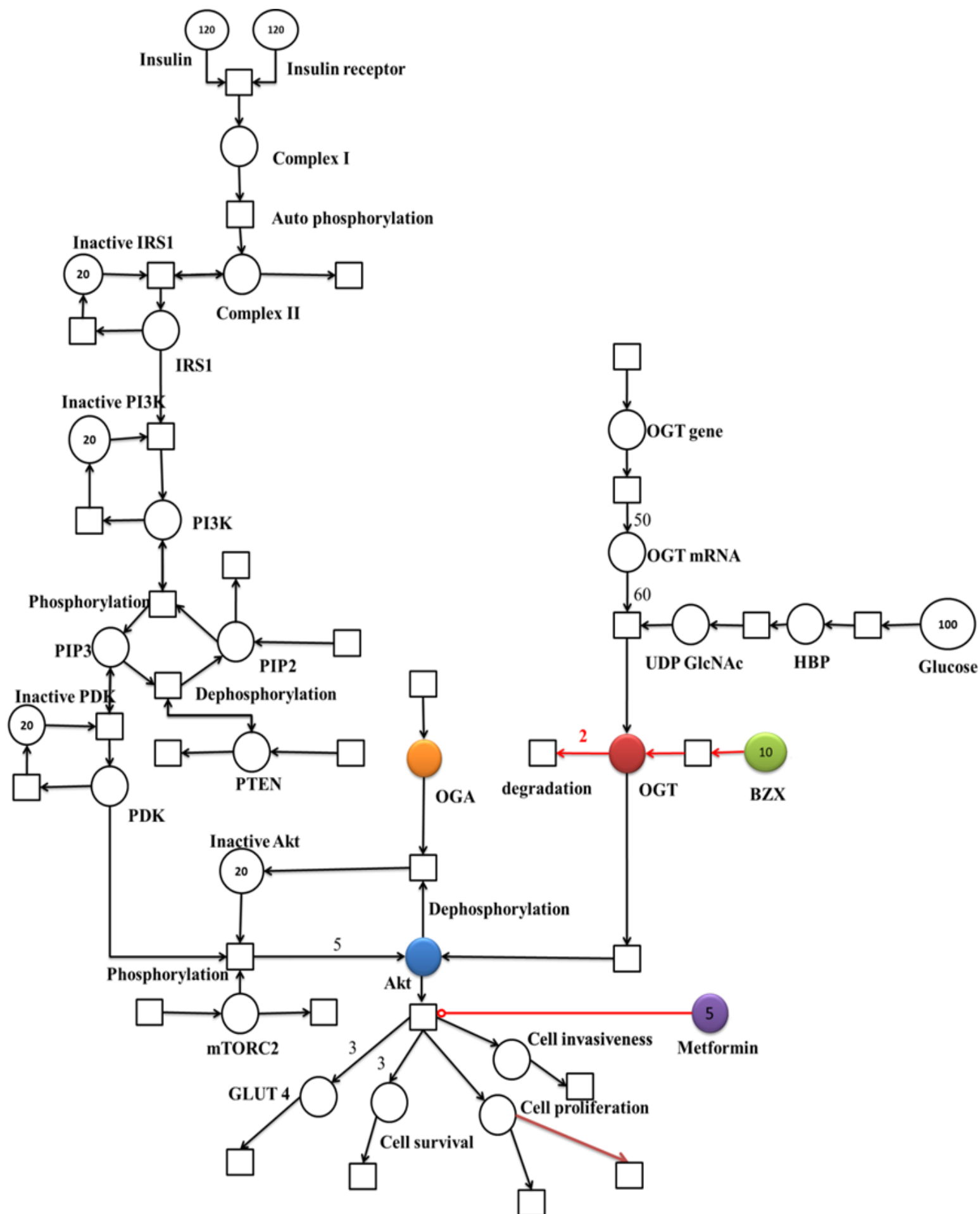

Supplement: Supplemental Information 6 — A standard place is illustrated as a circle representing proteins involved in the pathway. A continuous transition is depicted as square representing cellular processes including phosphorylation and dephosphorylation. A directed arc connects a place with a transition and vice versa. Inhibitory arc is represented by an arc with a hollow dot as its head in red color representing inhibition of cell proliferation by Metformin. Colored places include (Blue = Akt, Green = BZX, Red= OGT and Orange = OGA and Purple = Metformin). [file peerj-06-5917-s006.pdf]
